# Alterations of multiple peripheral inflammatory cytokine levels after repeated ketamine infusions in major depressive disorder

**DOI:** 10.1038/s41398-020-00933-z

**Published:** 2020-07-22

**Authors:** Yanni Zhan, Yanling Zhou, Wei Zheng, Weijian Liu, Chengyu Wang, Xiaofeng Lan, Xiurong Deng, Yan Xu, Bin Zhang, Yuping Ning

**Affiliations:** 1 The Affiliated Brain Hospital of Guangzhou Medical University (Guangzhou Huiai Hospital), Guangzhou, China; 2 The First School of Clinical Medicine, Southern Medical University, Guangzhou, Guangdong China; 3 Guangdong Engineering Technology Research Center for Translational Medicine of Mental Disorders, Guangzhou, China

**Keywords:** Predictive markers, Depression

## Abstract

Increasing evidence has demonstrated that inflammatory cytokines play an important role in major depressive disorder (MDD) and are associated with treatment outcomes. Few studies have explored the trajectories of multiple inflammatory cytokines after repeated ketamine infusions in MDD. In this study, we conducted a secondary analysis to investigate the impact of ketamine on the modulation of the inflammatory pathway in depression and whether this pathway contributes to the antidepressant properties of ketamine. A total of 60 patients with depression received six ketamine infusions (0.5 mg/kg) during a 12-day period. The Montgomery–Asberg Scale (MADRS) was administered, and blood samples were collected at baseline and 24 h and 14 days after the sixth infusion (days 0, 13, and 26). Plasma levels of the 19 cytokines were measured using the Luminex assay. At baseline, inflammatory cytokines were associated with the severity of depression. The concentrations of pro- and anti-inflammatory factors, including granulocyte macrophage colony-stimulating factor (GM-CSF), fractalkine, interferon gamma (IFN-γ), interleukin (IL)-10, IL-12p70, IL-17A, IL-1β, IL-2, IL-4, IL-23, IL-5, IL-6, IL-7, and tumor necrosis factor alpha (TNF-α), were downregulated after repeated ketamine administration (all *p* < 0.05). In addition, alterations in the levels of IL-17A (*r* = −0.259, *p* = 0.046) and IL-6 (*r* = −0.262, *p* = 0.043) were correlated with symptom improvement. A lower level of interferon-inducible T cell alpha chemoattractant (ITAC) at baseline was predictive of ketamine treatment response on day 13 according to a stepwise linear regression analysis (*β* = −0.296, *p* = 0.040). Our results suggest that the inflammatory pathway may be involved in the antidepressant effects of ketamine, which may be conducive to future treatment strategy optimization.

## Introduction

Major depressive disorder (MDD) is a devastating chronic mental disease that has increased disability and mortality worldwide and has a high rate of recurrence^1^. Treatment for this form of depression, including pharmacological and nonpharmacological treatment, is effective in relieving its symptoms to a large degree. However, more than one-third of MDD patients fail to achieve a response even after multiple treatment strategies are attempted^2^, and these patients are regarded as suffering from treatment-resistant depression (TRD), which indicates the urgent need to develop novel strategies to improve treatment outcomes.

Increasing evidence has demonstrated that immune dysregulation may play a role in pathophysiologic pathways involved in depression. The levels of pro-inflammatory cytokines, mainly tumor necrosis factor alpha (TNF-α), interleukin (IL)-6, and IL-1, in MDD are upregulated compared with those in healthy controls, which has been validated by previous meta-analyses^3,4^. Meanwhile, the levels of anti-inflammatory cytokines, including IL-4, IL-10, and IL-13, also show abnormalities in mood disorders^5,6^. In addition, the concentrations of other cytokines, such as IL-2, IL-5, IL-12, granulocyte macrophage colony-stimulating factor (GM-CSF), and interferon gamma (IFN-γ), are elevated in depression^7^. A recent study has indicated that antidepressant treatment is associated with significantly decreased levels of IL-1β and IL-6^8^. In addition, anti-inflammatory cytokines, such as IL-10, reversed depression-like behavior in a mouse model^9^. All of these findings suggest that inflammatory cytokines may be a vital mediator of depression and that an imbalance between pro- and anti-inflammatory cytokines contributes to depressive symptoms^10^.

Inflammatory hypotheses of depression have received growing attention in recent decades. Several mechanisms could underlie the relationship between cytokines and depression. In MDD, increases in pro-inflammatory cytokines, such as IL-6 and TNF-α, are related to hyperactivity of the hypothalamic–pituitary–adrenal axis, which consequently stimulates the secretion of corticotropin-releasing hormone and adrenocorticotropic hormone and secondarily stimulates the secretion of glucocorticoids^11,12^. In addition to glucocorticoid resistance, hypercortisolemia is found in depression^13^, which results in the dysfunction of immune-competent cells and adversely affects brain function^14^. In addition, cytokines are involved in monoamine and glutamatergic neurotransmission in the central nervous system (CNS). Pro-inflammatory cytokines are related to alteration of 5-hydroxytryptamine (5-HT) turnover in brain regions that results in a decreased level of 5-HT in the synaptic cleft, which may influence neuroplasticity and ultimately lead to depression^15,16^. Meanwhile, cytokine activity leads to increased expression of indoleamine-2,3-dioxygenase (IDO), which converts the 5-HT precursor tryptophan (TRP) to kynurenine (KYN) and further downregulates 5-HT synthesis^17^. Moreover, following the increase in pro-inflammatory cytokines, anti-inflammatory cytokines (e.g., IL-4 and IL-10) are elevated, which may occur in response to the upregulation of pro-inflammatory cytokines as an autoimmune response in MDD^18^. This increase in immune regulation could partially explain the spontaneous remission of depression^19^.

In recent decades, ketamine, a noncompetitive *N*-methyl-d-aspartate (NMDA) glutamate receptor antagonist, has been proven to improve depression rapidly when administered as a single subanesthetic dose^20–22^, and such therapeutic benefit can be amplified by adding additional infusions within the short term^23–25^. However, the mechanisms of the antidepressant efficacy of ketamine remain unclear. Several animal studies have shown that ketamine generates an anti-inflammatory effect that may contribute to its antidepressant action. In a study of a mouse model, chronic restraint stress exposure induced depressive-like behavior that was correlated with the upregulated expression of IL-1β, TNF-α, and IL-6, and the upregulation of these pro-inflammatory cytokines was reversed by ketamine infusions^26^. Other animal studies have revealed that ketamine administration downregulates the levels of IL-1β and IL-6 in the prefrontal cortex and hippocampus^27^ and decreases TNF-α and C-reactive protein levels in peripheral blood^28^; such immunoregulatory effects are primarily observed in microglia and eventually critically reduce quinolinic acid (QUIN) production in lipopolysaccharide-induced depression^29^. Regarding clinical research, our previous study showed that kynurenic acid (KYNA) levels and the KYNA/KYN ratio, which are regulated by inflammatory cytokines, were higher in ketamine responders than in non-responders, which indicates that the KYN pathway may be involved in the rapid antidepressant effect of ketamine^30^. In addition, a recent study demonstrated that a single ketamine infusion in patients with TRD could decrease the level of TNF-α, which was correlated with the antidepressant efficacy^31^. Another study showed that ketamine did not alter serum TNF-α levels but lowered serum IL-1β and IL-6 levels. This might be a predictive biomarker of the antidepressant action of ketamine^32^. In contrast, Park et al. found that ketamine administration increased IL-6 levels and that changes in cytokine levels were not associated with clinical improvement in patients with depression^33^. Although the results of previous studies varied, they indicate that inflammatory pathway modulation may contribute to the antidepressant efficacy of ketamine.

Changes in inflammatory cytokines after a single ketamine infusion in depression have been demonstrated previously^31,33,34^, while the effect on immunity of repeated infusions has been rarely reported. In this study, we conducted an open-label clinical trial of six ketamine infusions in patients with depression and examined multiple inflammatory cytokines throughout the treatment period. We aimed to investigate the impact of ketamine on the modulation of the inflammatory pathway in depression and whether this pathway mediates the antidepressant properties of ketamine. We hypothesized that pro-inflammatory cytokines (IL-1β, TNF-α, and IL-6) would be downregulated and anti-inflammatory cytokines (IL-4 and IL-10) would be increased after ketamine administration, and such alterations would be associated with depressive symptom improvement.

## Methods

This study was approved by the Clinical Research Ethics Committee of the Affiliated Brain Hospital of Guangzhou Medical University (Guangzhou Huiai Hospital) and was registered in the Chinese Clinical Trial Registry (ChiCTR-OOC-17012239). Outpatients or inpatients of the Affiliated Brain Hospital of Guangzhou Medical University were enrolled between November 2016 and July 2018. All the subjects clearly understood the research procedure and gave their written informed consent before the implementation of the study.

### Participants

All participants met the following criteria: (1) male or female patient aged 18–65 years; (2) had a diagnosis of MDD without psychotic symptoms according to the Diagnostic and Statistical Manual of Mental Disorders, fifth edition (DSM-V); (3) scored ≥17 on the 17-item Hamilton Depression Rating Scale^35^; (4) had a history of either TRD (history of nonresponse to two or more adequate antidepressant treatment trials) or suicidal ideation (Beck Scale for Suicide Ideation-part I ≥2); (5) able to understand and complete the study procedure; and (6) signed an informed consent form before participating in the program.

Subjects in the following circumstances were excluded: (1) diagnosed with bipolar depression (BD I/BD II); (2) had a current or previous diagnosis of any other severe mental disorder according to the Structured Clinical Interview for DSM-V (i.e., schizophrenia or organic mental disorders); (3) had a history substance abuse or dependence except for nicotine; (4) had a positive urine toxicological screening; (5) had a major medical illness (i.e., a serious and unstable physical disease, chronic inflammatory illness, or autoimmune disease); (6) was being treated with a combination of anti-inflammatory agents (i.e., nonsteroidal anti-inflammatory drugs or steroids); (7) was pregnant or was preparing for pregnancy or lactation; (8) was unable to carry out the study procedures.

### Study design and procedure

Enrolled participants received six intravenous infusions of ketamine over 2 weeks (treatment on Monday, Wednesday, and Friday). Ketamine hydrochloride (0.5 mg/kg) diluted in 0.9% saline (40 ml) was administered via infusion over 40 min 6 times in a 12-day period (days 1, 3, 5, 8, 10, and 12). During the infusion period, patients continued taking the same stable dosages of therapeutic medications they had received before the study. Additional details regarding the study procedure are given in our previous report^36^. Whole-blood samples were obtained at baseline and 24 h and 14 days after the sixth infusion (days 0, 13, and 26). The primary clinical outcome was the Montgomery–Asberg Depression Rating Scale (MADRS)^37^, which was administered at the same time points. Treatment response was defined as a ≥50% reduction in the MADRS score at 24 h after the final infusion (day 13) compared to the baseline MADRS score, and remission was indicated by MADRS total score ≤10.

### Measurement of cytokine levels

Blood samples were centrifuged at 3000 revolutions per minute at 4 °C for 10 min and frozen at −80 °C before further processing. The concentrations of 19 cytokines, including interferon-inducible T cell alpha chemoattractant (ITAC), GM-CSF, fractalkine, IFN-γ, IL-10, macrophage inflammatory protein (MIP)-3α, IL-12p70, IL-13, IL-17A, IL-1β, IL-2, IL-4, IL-23, IL-5, IL-6, IL-7, IL-8, MIP-1β, and TNF-α, were detected in plasma using a human high-sensitivity T cell magnetic bead panel (Millipore, Billerica, MA, USA, HSTCMAG-28SK) with a Luminex 200 multiplex immunoassay system based on the manufacturer’s instructions. Milliplex Analyst 5.1 software (EMD Millipore, Billerica, MA) was used with a cubic curve fitting to calculate the cytokine concentrations of the samples. All samples were run in duplicate, and the interassay coefficients of variability were <20%.

### Statistical analysis

Data were obtained from our previous open-label clinical trial^24^, sample size of which should be 92 to ensure a power of 90% and an *α* of 0.05 by using the PASS software (NCSS, USA). Patients who completed six infusions of ketamine and gave three blood samples were included in the final analysis. For the demographic and clinical characteristics, the continuous variables were described as the mean and standard deviation (SD), while the categorical variables were depicted as the frequency and percentage. For all the analytes, data were natural log-transformed prior to analysis. One-way analysis of variance was used to test the homogeneity of variances. Pearson’s bivariate correlation analysis was performed to explore the association between the baseline cytokine levels and depressive symptoms (measured by the MADRS score). Changes in cytokine levels and MADRS scores over time were examined using a linear mixed model, with age, gender, body mass index (BMI), major medical conditions, dose of antidepressant, and combined use of mood stabilizers/benzodiazepines/antipsychotics as covariates. Bonferroni correction was applied to post hoc comparisons for the time points examined. Afterward, the Benjamini–Hochberg method based on the false discovery rate was used for multiple comparisons between the 19 analytes. Spearman correlation analysis was used to assess the relationship between the changes in the ratio of depressive symptoms and inflammatory cytokines at each time point. To identify predictors of antidepressant response, stepwise linear regression analysis was conducted, in which baseline cytokines that significantly correlated with symptom improvement were independent variables while the reduction in the MADRS score was the dependent variable. All statistical analyses were performed using the Statistical Package for the Social Sciences version 20.0 (SPSS, Chicago, IL, USA). Significance was evaluated at *p* < 0.05 (two-tailed).

## Results

### Patient samples and characteristics

A total of 235 depressed patients were screened for eligibility, and 90 of them were successfully enrolled. These patients then received six ketamine infusions, and 81 of the patients were followed up for 2 weeks after the infusions (day 26). As all three required blood samples were partially absent for 21 patients, data were collected from the remaining 60 patients from whom all three blood samples had been obtained. For the entire cohort, 36.7% were male (*N* = 22), and the mean age was 34.45 ± 11.92 years. The mean baseline MADRS score was 32.22 ± 7.96 points (Table 1).

**Table 1:** Baseline demographic and clinical characteristics.

Variables	Total sample (*n* = 60)	
	*N*	%
Male sex	22	36.7%
Married	30	50.0%
Employed	24	40.0%
Living alone	5	8.3%
History of psychiatric hospitalization	12	20.0%
Family history of psychiatric disorders	16	26.7%
Major medical condition(s)	12	20.0%
Current smoking	6	10.0%
Current drinking	1	1.7%
>1 antidepressant	9	15.0%
On mood stabilizer	12	20.0%
On benzodiazepine	31	51.7%
On antipsychotic	37	61.7%
	**Mean**	**SD**
Age (years)	34.45	11.92
Education (years)	12.35	3.28
BMI (kg/m^2^)	22.35	3.35
Age of onset (years)	28.30	11.83
Duration of illness (months)	70.10	61.25
FLUeq	39.83	22.17
CPZeq	106.18	120.08
Baseline MADRS total score	32.22	7.96

*BMI* body mass index, *FLUeq* fluoxetine equivalents milligrams, *CPZeq* chlorpromazine equivalent milligrams, *MADRS* Montgomery–Asberg Depression Rating Scale.

### Inflammatory cytokines and depressive symptoms

The natural log-transformed values for the 19 inflammatory cytokines at baseline are shown in Table 2. The baseline MADRS score was positively correlated with the baseline levels of IL-10 (*r* = 0.268, *p* = 0.039), IL-12p70 (*r* = 0.365, *p* = 0.004), IL-13 (*r* = 0.324, *p* = 0.012), IL-17A (*r* = 0.297, *p* = 0.021), IL-1β (*r* = 0.270, *p* = 0.037), IL-2 (*r* = 0.297, *p* = 0.021), IL-23 (*r* = 0.310, *p* = 0.016), IL-5 (*r* = 0.282, *p* = 0.029), IL-6 (*r* = 0.300, *p* = 0.020), and IL-7 (*r* = 0.297, *p* = 0.021) and negatively correlated with the baseline level of MIP-1β (*r* = −0.290, *p* = 0.025). The levels of other cytokines were not significantly associated with depressive symptoms.

**Table 2:** Correlation of the MADRS scores and level of cytokines at baseline.

Variables	Concentration	MADRS scores	
	Mean ± SD	*r*	*p*
ITAC	1.12 ± 0.25	−0.001	0.996
GM-CSF	1.66 ± 0.36	0.200	0.125
Fractalkine	2.49 ± 0.25	0.234	0.072
IFN-γ	0.99 ± 0.24	0.087	0.507
IL-10	1.14 ± 0.50	0.268	**0.039**
MIP-3α	1.08 ± 0.35	0.181	0.166
IL-12p70	0.38 ± 0.35	0.365	**0.004**
IL-13	0.54 ± 0.38	0.324	**0.012**
IL-17A	0.91 ± 0.29	0.297	**0.021**
IL-1β	0.09 ± 0.34	0.270	**0.037**
IL-2	0.51 ± 0.33	0.297	**0.021**
IL-4	1.47 ± 0.24	0.205	0.122
IL-23	2.32 ± 0.45	0.310	**0.016**
IL-5	0.70 ± 0.43	0.282	**0.029**
IL-6	0.22 ± 0.34	0.300	**0.020**
IL-7	0.91 ± 0.34	0.297	**0.021**
IL-8	0.37 ± 0.26	0.208	0.112
MIP-1β	1.14 ± 0.33	−0.290	**0.025**
TNF-α	0.57 ± 0.17	0.106	0.418

All concentrations are presented as natural log-transformed (pg/ml).

*ITAC* interferon-inducible T cell alpha chemoattractant, *GM-CSF* granulocyte macrophage colony-stimulating factor, *IFN-γ* interferon gamma, *IL* interleukin, *MIP* macrophage inflammatory protein, *TNF-α* tumor necrosis factor alpha, *MADRS* Montgomery–Asberg Depression Rating Scale.

Bolded values are *p* < 0.05.

### Treatment response and inflammatory cytokine alterations after ketamine infusions

**Fig. 1:**
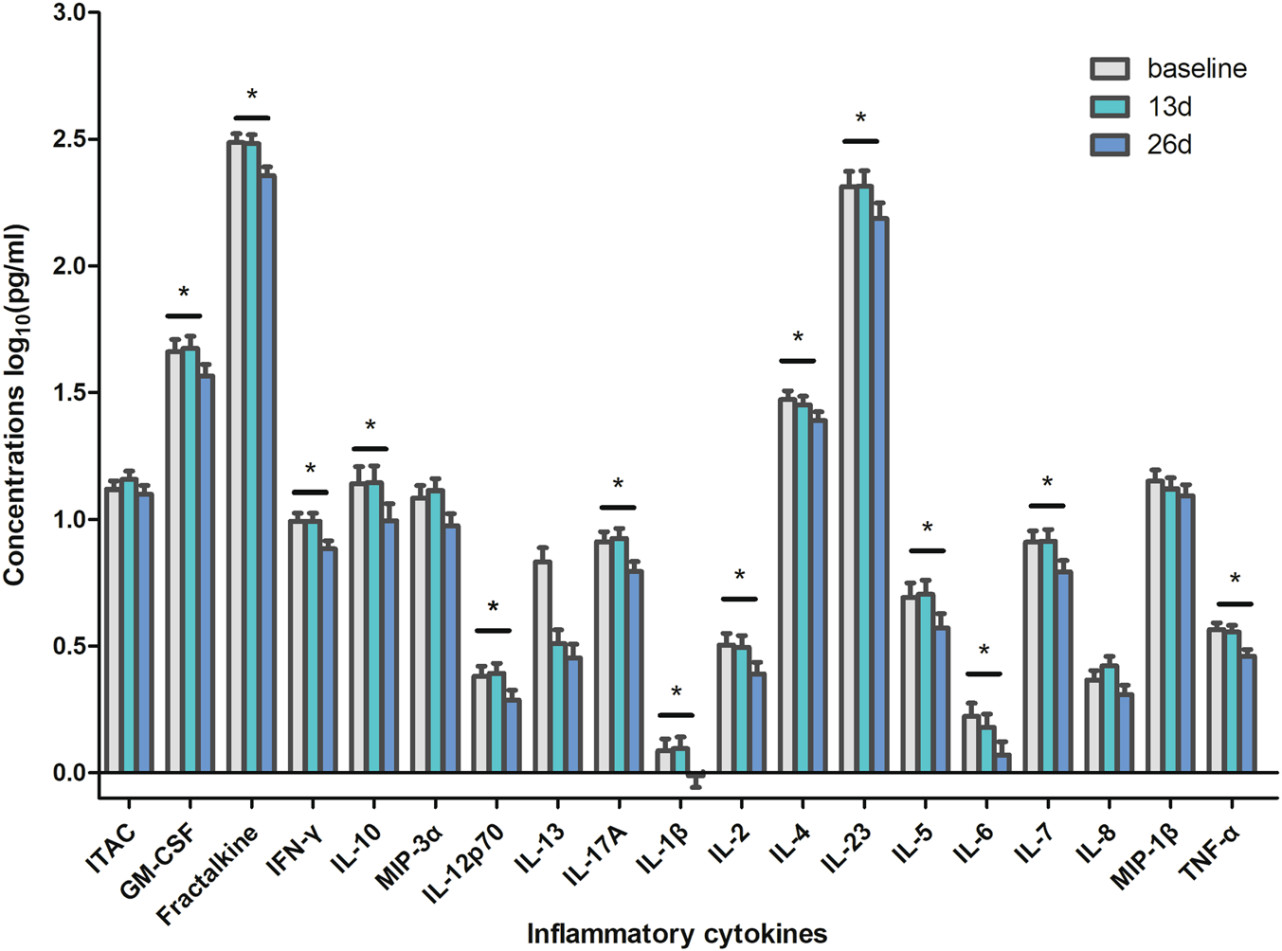
Change in inflammatory cytokines in MDD before and after treatment with ketamine. ITAC interferon-inducible T cell alpha chemoattractant, GM-CSF granulocyte macrophage colony-stimulating factor, IFN-γ interferon gamma, IL interleukin, MIP macrophage inflammatory protein, TNF-α tumor necrosis factor alpha. Asterisk (*) represents significant difference between baseline and day 26 (*p* < 0.05).

Ultimately, 35 (58.3%) patients met the response criteria after six ketamine infusions (day 13), and 25 (41.7%) achieved remission. Linear mixed models were employed to explore the changes in clinical symptoms and inflammatory cytokine levels over time. A significant decrease in the MADRS scores was found on day 13 and day 26 compared to the baseline scores. For the 19 cytokines that were examined, no significant changes were found between baseline and day 13. However, there were 14 inflammatory cytokines that differed significantly at 2 weeks postinfusion (day 26). After controlling for covariates of BMI, age, gender, major medical conditions, dose of antidepressant, and combined use of mood stabilizers/benzodiazepines/antipsychotics, the levels of GM-CSF, fractalkine, IFN-γ, IL-10, IL-12p70, IL-17A, IL-1β, IL-2, IL-4, IL-23, IL-5, IL6, IL-7, and TNF-α were significantly lower on day 26 than at baseline (Table 3 and Fig. 1).

**Table 3:** Comparison of plasma cytokines after six ketamine infusions using linear mixed-model analysis.

Variables	*F*	*p*	Day 13 vs day 0		Day 26 vs day 0	
			*t*	*p*	*t*	*p*
MADRS	111.313	**<0.001**	12.821	**<0.001**	12.802	**<0.001**
ITAC	1.430	4.617	−1.186	0.725	0.438	1.000
GM-CSF	6.123	**0.007**	−0.389	1.000	2.857	**0.025**
Fractalkine	13.211	**<0.001**	0.138	1.000	4.517	**<0.001**
IFN-γ	7.759	**0.001**	0	1.000	3.375	**0.005**
IL-10	7.475	**0.001**	−0.091	1.000	3.364	**0.005**
MIP-3α	6.111	**0.007**	−0.683	1.000	2.634	0.052
IL-12p70	8.557	**0.001**	−0.333	1.000	3.133	**0.011**
IL-13	2.341	0.855	0.657	1.000	2.171	0.182
IL-17A	10.325	**<0.001**	−0.375	1.000	3.688	**0.002**
IL-1β	5.841	**0.007**	−0.229	1.000	2.886	**0.028**
IL-2	7.467	**0.001**	0.273	1.000	3.424	**0.004**
IL-4	4.034	0.095	0.700	1.000	2.800	**0.044**
IL-23	5.813	**0.007**	−0.048	1.000	2.977	**0.022**
IL-5	6.993	**0.001**	−0.282	1.000	3.128	**0.016**
IL-6	6.157	**0.007**	0.933	1.000	3.378	**0.007**
IL-7	8.438	**<0.001**	−0.152	1.000	3.485	**0.005**
IL-8	4.941	**0.034**	−1.571	0.482	1.543	0.992
MIP-1β	2.819	0.526	1.250	0.956	2.292	0.214
TNF-α	9.89	**<0.001**	0.385	1.000	4.000	**<0.001**

*ITAC* interferon-inducible T cell alpha chemoattractant, *GM-CSF* granulocyte macrophage colony-stimulating factor, *IFN-γ* interferon gamma, *IL* interleukin, *MIP* macrophage inflammatory protein, *TNF-α* tumor necrosis factor alpha, *MADRS* Montgomery–Asberg Depression Rating Scale.

Bolded values are *p* < 0.05.

Spearman correlation analysis showed that the reduction in the MADRS scores following the ketamine infusions was associated with changes in IL-17A (*r* = −0.259, *p* = 0.046) and IL-6 (*r* = −0.262, *p* = 0.043) on day 13 but was no longer related to any factor on day 26.

### ITAC as a predictor of ketamine treatment response

The baseline level of ITAC was related to a reduction in the MADRS score on day 13 (*r* = −0.276, *p* = 0.033). In the stepwise regression analysis model, the baseline value of ITAC was negatively associated with symptom improvement (*β* = −0.276, *p* = 0.033). After controlling for multiple covariates, ITAC remained a significant predictor of treatment response (*β* = −0.296, *p* = 0.040; Table 4).

**Table 4:** Stepwise linear regression of reductions in MADRS scores on day 13 and level of cytokine at baseline.

Dependent variable	Independent variable	*B*	S.E.	*β*	*t*	*p*	95% CI
Reduction in MADRS on day 13	ITAC	−12.557	5.95	−0.296	−2.110	0.040	−24.508 to −0.606

*ITAC* interferon-inducible T cell alpha chemoattractant, *MADRS* Montgomery–Asberg Depression Rating Scale, *S.E.* standard error, *CI* confidence interval.

There were two blocks of independent variables in this linear regression model, the first block included age, gender, BMI, dose of antidepressant, and combined use of mood stabilizer/benzodiazepine/antipsychotic, and the second block included cytokines that were significantly correlated with reduction in MADRS on day 13.

## Discussion

To the best of our knowledge, this secondary analysis is the first to explore multiple inflammatory cytokines after repeated ketamine infusions in patients with MDD. The results are partially consistent with the proposed hypothesis. Several salient findings were obtained. First, we found that the baseline levels of IL-10, IL-12p70, IL-13, IL-17A, IL-1β, IL-2, IL-23, IL-5, IL-6, IL-7, and MIP-1β were associated with depression symptoms. Second, the levels of inflammatory cytokines, including GM-CSF, fractalkine, IFN-γ, IL-10, IL-12p70, IL-17A, IL-1β, IL-2, IL-4, IL-23, IL-5, IL-6, IL-7, and TNF-α, tended to be downregulated after six ketamine infusions and were significantly lower on day 26. Changes in the levels of IL-17A and IL-6 were correlated with symptom improvement on day 13. Finally, we identified for the first time that lower levels of ITAC at baseline could be predictive of ketamine treatment response.

### Cytokines and treatment response

Our study showed that the baseline plasma cytokine levels were significantly correlated with symptom severity, which is consistent with the results of previously reported studies^38,39^. Abundant evidence has shown that the levels of inflammatory cytokines are higher in MDD patients than in healthy controls^6,40^. Our results are robust evidence of the relationship between inflammatory dysregulation and depression.

In line with our expectations, the levels of the pro-inflammatory cytokines TNF-α, IL-1β, and IL-6 were decreased after the ketamine infusions in our study, which is consistent with the results of previous studies^31–34^ that administered a single infusion in patients with depression. We found that changes in the IL-6 and IL-17A levels were significantly negatively associated with the antidepressant response. Previous studies indicated that IL-6 and IL-17A are related to depressive symptoms^3,41^. The relationship between these cytokines and antidepressant effects is complex and has been reported inconsistently. Yoshimura et al. showed that IL-6 changes are positively correlated with depressive symptom improvement^42^, while other research demonstrated that such changes are negatively associated with symptom improvement after paroxetine treatment in MDD^43^. Moreover, a significant correlation between changes in IL-6 levels and depressive symptoms was negative in males but positive in females following venlafaxine treatment^43^, which suggests the existence of gender differences in the mechanism of antidepressant action. Regarding ketamine infusion, Park et al. and Kiraly et al. found that the alteration of cytokine levels is not associated with a rapid antidepressant response to ketamine^33,34^. These inconsistent findings are probably due to differences in treatment protocols, detection timing, and study samples (patients with TRD and suicidal ideation in our study vs patients with only TRD in Kiraly’s study). In addition, the fact that the participants in this study were younger, with a shorter duration of illness, and higher doses of medication (especially antipsychotics) compared to those in Park’s study is a contributor to the conflicting results.

Preclinical studies have shown that ketamine acts in the inflammatory system, which may contribute to the antidepressant efficacy. For example, Reus et al. reported that the levels of TNF-α, IL-1, and IL-6 were increased in the serum and cerebrospinal fluid of rats with maternal deprivation-induced depressive-like behavior. Ketamine treatment at a dosage of 15 mg/kg for 14 days reversed the changes in both cytokine levels and behavior^44^. In addition to the attenuation of depressive-like behavior, significantly decreased expression of TNF-α, IL-1β, and IL-6 in the prefrontal cortex and hippocampus of a rat model was found following administration of 10 mg/kg ketamine^27,45^. These animal studies suggest that the antidepressant effects of ketamine may involve the regulation of pro-inflammatory cytokines in both peripheral and central regions, even though the mechanism has not yet been clearly identified. One possible pathophysiology involved in depression is the triggering of glutamatergic system dysregulation by cytokines. Increasing the levels of pro-inflammatory cytokines could stimulate astrocytes to release glutamate directly^46^. Meanwhile, immune activation could also cause IDO, a validated NMDA receptor agonist, to convert TRP to KYN. KYN is subsequently metabolized into 3-OH-kynurenine, which then activates microglia to produce QUIN. QUIN is an NMDA receptor agonist that can upregulate glutamate release and has neurotoxic effects^17^. The resulting glutamate excitotoxicity further activates presynaptic NMDA receptors and leads to depressive symptoms^17^. Given that ketamine is an NMDA receptor antagonist, it may exert its antidepressant effect by blocking the above described inflammatory pathway. In addition, ketamine is mainly metabolized into hydroxynorketamines in the plasma in humans^47^, which is associated with enhanced production of alpha-amino-3-hydroxy-5-methyl-4-isoxazolepropionic acid^48^. In addition, the rapamycin (mammalian target of rapamycin) pathway is activated by ketamine, resulting in the increased activity of new spine synapses^49^. These pathways may synergistically contribute to the antidepressant effect of ketamine.

To our surprise, as the levels of pro-inflammatory cytokines decreased after the ketamine infusions, the levels of anti-inflammatory cytokines such as IL-4 and IL-10 also showed decreased expression according to our results, which is contrary to our hypothesis. Previous meta-analyses have suggested that not only pro-inflammatory cytokines but also IL-10, an anti-inflammatory cytokine, are significantly elevated in MDD patients compared to those in healthy controls^6,50^, which indicates that a more complicated mechanism is involved in depression. Maes et al. reported that the immune-inflammatory response system (IRS) was activated in MDD, as reflected by the increased levels of pro-inflammatory cytokines, which consequently induce the compensatory immune-regulatory reflex system (CIRS). The CIRS pathway was associated with the elevation of T helper type 2 and T regulatory activities, resulting in increased levels of IL-4 and IL-10, which may further downregulate IRS and contribute to the immune pathophysiology of MDD^19,51^. The existence of the CIRS could protect against an excessive immune system response and restore a balanced state. Therefore, the decline in anti-inflammatory cytokine levels after ketamine infusions may be a result of the weakening of the CIRS pathway caused by decreased IRS activity. Notably, a recent meta-analysis of 32 studies that examined changes in peripheral markers in MDD revealed a significant decrease in IL-4 and IL-10 levels after antidepressant treatment^52^. therefore the combined antidepressant use in the present study may have confounded the results.

In addition, unlike the depressive symptom response, which was observed after six ketamine infusions on day 13, inflammatory cytokine levels did not show a significant decrease until day 26. The potential explanation is that the antidepressant effect of ketamine may not be mediated by the inflammatory pathway. Park et al. showed that cytokines may not be valid biomarkers of the antidepressant effect of ketamine^33^. However, our study found that the antidepressant effect was correlated with changes in IL-6 and IL-17A levels. Considering the use of a single infusion in the former study and the use of repeated infusions in the latter study, it is difficult to draw a definitive conclusion. Another mechanism involves the possible existence of a delayed response in peripheral inflammatory cytokines like that in the CNS after ketamine infusions. Previous studies have shown that the response of inflammatory cytokines is out-of-sync with depressive symptoms. Readler et al. demonstrated that the levels of inflammatory cytokines were still high even if depressive symptoms were relieved^53^. In addition, a recent meta-analysis also showed that sIL-1RA may remain increased after the relief of depression^50^. Moreover, the IRS and CIRS pathways are active even in the remission stage of a mood disorder, which suggests that the original steady state may not be restored immediately after an acute episode^19^. The mechanism of this desynchrony is not yet fully understood and needs further exploration.

### Predictors of treatment response

The rapid antidepressant effect of ketamine has been demonstrated in our study and previous studies^54,55^. However, psychotomimetic, cardiovascular, and neurological side effects after acute ketamine treatment could limit its clinical utility^56^. The development of biomarkers to predict treatment outcome will be beneficial for personalizing medicine. Previously reported studies have shown that higher peripheral levels of Shank3 and IL-6 as well as lower levels of adiponectin at baseline could predict antidepressant efficacy after administration of a single ketamine dose^32,57,58^. Our previous study showed that changes in KYN pathway metabolite levels were correlated with the treatment response after repeated infusions^30^. In this study, we first found that lower baseline levels of ITAC were predictive of symptom improvement following six ketamine administrations. ITAC is regarded as an important chemokine for the recruitment of T cells in inflammation and the enhancement immune responses, which have been well studied in inflammatory disease but poorly examined in depression^59^. The mechanism of ITAC in depression remains unclear and requires further study.

## Limitations

This study has several limitations. First, a control group was not used due to the goal of exploring precise treatment for depression of our study, which limits the analysis of efficacy. In addition, the absence of a control group meant that we could not control for the natural fluctuations in cytokine levels over time. However, a previous study has shown that the levels of multiple cytokines, including most of the cytokines examined in our study, are stable over a short time period^60^. A randomized, double-blind, and controlled trial should be conducted in the future to verify the current results. Second, all the patients maintained their prior medication regimen during the ketamine infusions due to ethical reasons and to better reflect the situation in the “real world”. Such combined antidepressant use may play a part in the regulation of the immune system. In addition, conditions such as concurrent depression treatment (e.g., supplements and exercise) and chronic pain may exert a potential effect on cytokines. Hence, the alteration of inflammatory cytokines in our study could be properly explained as an add-on effect of repeated ketamine infusion. Finally, we measured the analytes in the periphery rather than in the central regions. Whether the regulation of inflammatory cytokines in the CNS involves the same pathway as that in the periphery has been questioned, which requires further clarification.

## Conclusion

Our study has obtained robust evidence that inflammatory cytokines are associated with symptom severity in MDD. Inflammatory cytokines were downregulated after repeated ketamine administration, and such alterations were correlated with depressive symptom improvement. In addition, a decreased level of ITAC at baseline could be predictive of the antidepressant response to ketamine. Overall, our findings suggest that the inflammatory pathway may be involved in the antidepressant effects of ketamine, which may contribute to future treatment strategy optimization.
